# The effect of transcutaneous spinal cord stimulation on the balance and neurophysiological characteristics of young healthy adults

**DOI:** 10.1017/wtc.2023.24

**Published:** 2024-02-08

**Authors:** Isirame Omofuma, Robert Carrera, Jayson King-Ori, Sunil K. Agrawal

**Affiliations:** 1 Mechanical Engineering Department, Columbia University, New York, NY, USA; 2 Deployment Roboticist, Covariant AI, Emeryville, CA, USA

**Keywords:** rehabilitation robotics, postural control, transcutaneous spinal cord stimulation, cable driven devices

## Abstract

Transcutaneous spinal cord stimulation (TSCS) is gaining popularity as a noninvasive alternative to epidural stimulation. However, there is still much to learn about its effects and utility in assisting recovery of motor control. In this study, we applied TSCS to healthy subjects concurrently performing a functional training task to study its effects during a training intervention. We first carried out neurophysiological tests to characterize the H-reflex, H-reflex recovery, and posterior root muscle reflex thresholds, and then conducted balance tests, first without TSCS and then with TSCS. Balance tests included trunk perturbations in forward, backward, left, and right directions, and subjects’ balance was characterized by their response to force perturbations. A balance training task involved the subjects playing a catch-and-throw game in virtual reality (VR) while receiving trunk perturbations and TSCS. Balance tests with and without TSCS were conducted after the VR training to measure subjects’ post-training balance characteristics and then neurophysiological tests were carried out again. Statistical comparisons using t-tests between the balance and neurophysiological data collected before and after the VR training intervention found that the immediate effect of TSCS was to increase muscle activity during forward perturbations and to reduce balance performance in that direction. Muscle activity decreased after training and even more once TSCS was turned off. We thus observed an interaction of effects where TSCS increased muscle activity while the physical training decreased it.

## 1. Introduction

Transcutaneous spinal cord stimulation (TSCS) is a noninvasive neuromodulation technique that can be used to excite sensory neurons in the posterior root of the spinal column. The effects of TSCS are similar to those of epidural stimulation (ES), which targets the same spinal structures (Hofstoetter et al., 2018; García et al., 2019) and has been shown to be effective in restoring motor function in spinal cord injury (SCI) patients (Rejc et al., 2015; Eisdorfer et al., 2020; Oh et al., 2021). However, electrode implantation in ES comes with the risks and expenses of invasive surgery and possible infection after surgery. TSCS poses a lower risk as stimulation is provided on the surface of the skin and therefore could make this treatment more accessible.

There is still much to be understood about the mechanisms by which electrical stimulation affects the nervous system and the interaction of the many stimulation parameters, such as stimulation frequency, current amplitude, and so forth, that affect the outcomes of the interventions (Oh et al., 2021). We investigate in this study the effects on balance and neurophysiological measures when TSCS is applied to participants performing a functional balance task.

In a previous study, we examined the effects of applying only TSCS on the neurophysiological and balance characteristics of a group of healthy adults (Carrera et al., 2022). We found that although we observed an increase in spinal excitability when applying TSCS, it did not lead to improved performance of the functional balance task. This reinforces a frequently emphasized principle in the literature that functional training is essential for neurophysiological changes to translate into improved function (Taube et al., 2007; Kleim and Jones, 2008; Nahum et al., 2013; Flores et al., 2021).

In a study involving cervically spinalized rats by Girgis et al. (2007), rats were divided into two groups. One group was trained to perform a reaching task for 6 weeks immediately after injury, while the other group did not perform reach training. The study showed that the group of rats that underwent functional reach training improved in the reaching task substantially when compared to the untrained group. In another study, Flores et al. (2021) showed that in the presence of the neuroplasticity boosting effects of Chondroitinase ABC (Mahajan, 2018), rats that underwent specific functional rehabilitation demonstrated considerable improvement in the reaching task when compared to rats that received only Chondroitinase. These studies demonstrate that activity-based training can lead to improved functions.

García et al. (2019) note that spinal stimulation interventions must be combined with activity-based rehabilitation to evoke adaptive neuroplasticity. This includes movement, strength, coordination, walking, manual dexterity, and grip strength exercises. The mechanism by which physical training benefits recovery of motor function in SCI is not well understood (Lynskey, 2008; Flores et al., 2021), however, there are hypotheses in the literature that support activity-based training. Lynskey (2008) suggests that recovery after activity-based training relies on sensory feedback mechanisms. Fouad and Tetzlaff (2012) present a number of motor recovery mechanisms that have been observed with activity-based rehabilitation. These include increase in the presence of growth chemicals like the brain-derived neurotrophic factor (BDNF), changes to inhibition within the spinal circuitry, changes in spinal excitability, and changes in neuronal properties. Their study also notes a strong relation between neuronal activity and plasticity. Since both functional training and electrical stimulation aim at changes in neuronal activity, they can both potentially create the conditions for recovery.

Based on this knowledge, we hypothesize that functional training and TSCS applied simultaneously will lead to gains in functional performance along with increased spinal excitability. We have designed an experiment to assess how TSCS affects balance performance in healthy subjects under the following conditions: (i) immediately after TSCS is applied and (ii) after a perturbation-based functional training intervention with simultaneous application of TSCS. In a recent experiment, Meyer et al. (2020) showed that TSCS immediately improves motor function in incomplete SCI when applied. Hence, we hypothesize that TSCS will increase motor function performance both immediately after it is turned on and after the training intervention.

## 2. Methods

### 2.1. Experimental protocol—study design

**Figure 1.**
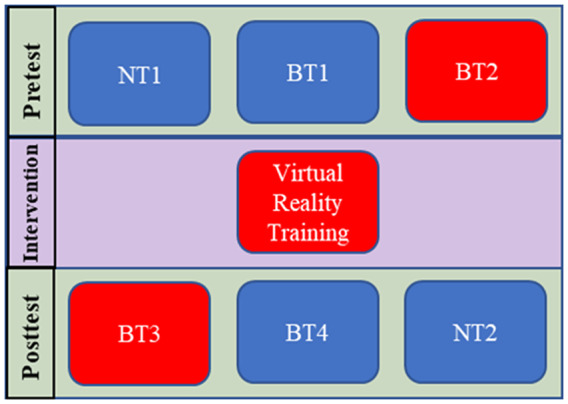
Experimental test plan. Boxes in blue are sessions that were conducted without TSCS stimulation and those in red were with TSCS. BT, balance test; NT, neurophysiological test.

Study protocols and consent forms were approved by Columbia University’s Institutional Review Board (IRB-AAAR6780). The experimental structure in Figure 1 shows the protocol used in this one-day experiment to measure the immediate effect of TSCS and its effect after combining it with a training protocol. Neurophysiological tests NT1 and NT2, described in Section 2.1.1, bookended the experiment and measure the spinal excitability at the beginning and at the end, respectively. NT1 was followed by the first balance test, BT1, conducted without TSCS and then by the second balance test, BT2, conducted with TSCS (Section 2.1.2). After these pretests, the subjects were exposed to repeated perturbations in a catch-and-throw virtual reality game to train their reactive postural control. TSCS was used during the training intervention. Post-training tests BT3, with TSCS, and BT4, without TSCS, followed the VR training session and lastly, NT2 was carried out. NT1 and NT2 both took about 30 min, while each balance test took about 15 min. The VR training session took about 30 min and transitioning between each test took 5–10 min.

**Figure 2.**
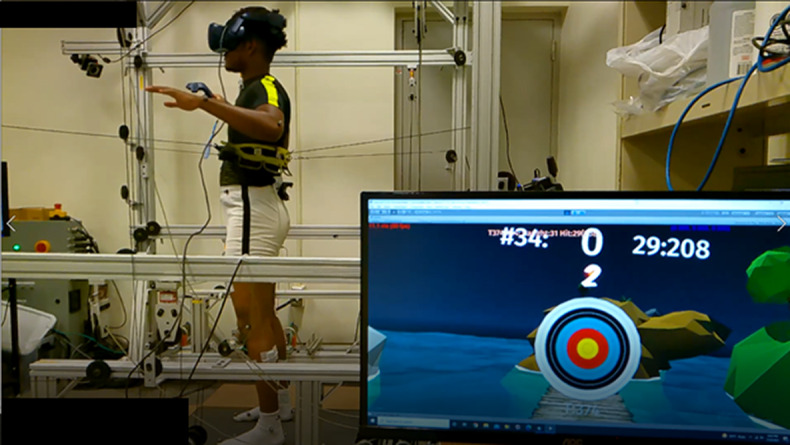
Subject standing in RobUST. The subject stands in the middle of the frame and a belt controlled by motors, through cables, is connected to their trunk. The subject wears a VR headset which displays a scene shown at the bottom right. The subject plays a VR catch-and-throw game using the VR wand for the catch and throw actions while receiving perturbations.

Eleven subjects were enrolled in this study: 7 male (age: 25.8 ± 4.8 years; weight: 75 ± 6 kg; height: 1.79 ± 0.08 m) and 4 female (age: 22.8 ± 4.4 years; weight: 67.9 ± 16.5 kg; height: 1.64 ± 0.05 m).

#### 2.1.1. Neurophysiological pretest (NT1)

The experiment began with the collection of neurophysiological metrics. The ascending limb of the H-reflex (Palmieri et al., 2004; Knikou, 2008), the H-reflex recovery cycle (Masland, 1972), and the posterior root muscle (PRM) reflex threshold (Hofstoetter et al., 2018) were measured. All neurophysiological tests were conducted while subjects lay supine.

We measured each subject’s H-reflex response only over its ascending limb to optimize experimentation time. The cathode was placed on the midline of the popliteal fossa to stimulate the tibial nerve, and the anode, just above the patella. Subjects were stimulated with a 1 ms monophasic pulse starting at 5 mA. The stimulating current was increased progressively by 5 mA until the maximum H-reflex response, H-max, was reached. Electromyography (EMG) recording of the H-reflex was recorded with a wireless EMG sensor (Delsys Trigno, Natick, MA) placed on the soleus muscle and streamed to a custom MATLAB program for real-time processing. After collecting data on the ascending limb, the H-reflex current for generating 30% of H-max was determined and used when evaluating H-reflex recovery (Carrera et al., 2022).

H-reflex recovery was measured with five sets of paired pulses at 80, 150, 250 ms, 500, and 1,000 ms interstimulus intervals (ISIs). The pulses had the same profile as that for eliciting H-reflexes but, to measure recovery, two pulses were applied in quick succession at the ISIs listed. Doing this generates two H-reflex responses, the second, conditioned response, is smaller than the first. Reduction in the size of the H-reflex is hypothesized in the literature to be due to depletion of electrolytes, spinal circuit inhibition, or other neuro-physiological processes. We use this as a measure of the state of a subject’s spinal excitability (Misiaszek, 2003). Paired pulses were applied 15 s apart with a current approximately 30% of H-max. This was determined from the ascending limb of the H-reflex recruitment curve after a sigmoid fitting using measurements taken when characterizing the H-reflex (Klimstra and Zehr, 2007).

Posterior root muscle (PRM) reflex thresholds were generated using a stimulating electrode (cathode) placed mid-line on the subject’s back between the L2–L3 vertebrae. The cathode was placed just above the umbilicus. For all PRM stimulations, a 500 μs pulse width, monophasic signal was used. The PRM threshold was determined with the following procedure:

1. Start a single pulse stimulation at 30 mA and progressively increase current by 5 mA until a response in the left or right soleus is recorded with magnitude above 50 μV. The current at which this happens is noted.
2. Reduce current by 1 mA until the PRM responses generated are below 50 μV.
3. At this point, increase current again by 1 mA until the response is again above 50ÂμV. This current is taken as the PRM threshold.

Once this threshold was found, subjects were stimulated eight times at 120% of the threshold value. The response at this level was compared between NT1 and NT2.

#### 2.1.2. Balance test 1 (BT1)

Balance was assessed by perturbing subjects at the trunk while they stood upright in the Robotic Upright Stand Trainer (RobUST), a robotic testing device developed in the Columbia University ROAR Lab (Khan et al., 2019; Luna et al., 2020). RobUST is a cable-driven exoskeleton capable of applying forces to a subject through a brace worn around the trunk that is attached to four cables. Each cable is attached to a motor, and with this configuration, the device can apply forces in the horizontal plane. RobUST was used to perturb subjects at the trunk in the forward, backward, right, and left directions in this experiment.

Once a participant was set up in the device, the subject’s perturbation threshold was found in each perturbation direction. Subjects were perturbed twice starting at 25% body weight (BW) in each direction. If they did not lose balance both times, the force was increased by 5% BW. We defined the perturbation threshold as the perturbation force such that 5% above this value, the subject failed to maintain balance twice in a row. This value was used in all subsequent balance tests.

In each balance test, subjects were tested 10 times in each direction at the perturbation force threshold. The sequence of directions in which the subjects were perturbed was randomized in four directions, that is, forward, backward, left, and right. Successful trials were defined as those in which the subject stayed in place without taking a step or reaching for external support after being perturbed. Perturbations had a trapezoidal profile in which a controller commanded cable force to rise from a base level to the target force level over 75 ms, maintain that constant force for 400 ms, and then decay to zero over 100 ms, with a total pulse time of 575 ms.

#### 2.1.3. Setting up TSCS

After BT1, a stimulating cathode was placed on the subject’s back, midline between the L2-L3 vertebrae, while the anode was placed midline just above the umbilicus as the subject stood in RobUST (Figure 2). Continuous TSCS in this experiment consisted of a continuous train of monophasic pulses, at a frequency of 30 Hz, and pulse width of 500 μs. Subjects were initially stimulated with 5, 7.5, and 10 mA amplitude continuous TSCS and were asked to identify the highest stimulating current they could tolerate. This chosen current level was then used for all subsequent TSCS stimulations of the subject. Further description of the application of TSCS is provided in Carrera et al. (2022).

#### 2.1.4. Balance test 2 (BT2)

Perturbations in BT2 were carried out as in BT1. TSCS was started at the beginning of this session and remained on throughout the test. While receiving TSCS, force perturbations were applied to subjects as in BT1.

#### 2.1.5. Virtual reality training with TSCS

After the two balance tests, the subjects played a catch-and-throw virtual reality game using an HTC Vive VR system. The game consisted of the subjects catching a fireball shot at them from a distance using the VR system wand. The ball traveled to the subject over 1 s and perturbations were consistently applied 800 ms after the ball was shot at the subject so that they were perturbed just when they were about to catch the ball. The subjects went through two sessions of 50 catch-and-throw trials while still receiving TSCS. Subjects were asked to take a break for 5 min before and after the VR sessions. This activity provided the opportunity for subjects to improve their balance control through repeated practice of balance restoration strategies.

#### 2.1.6. Balance test 3 and 4 (BT3 and BT4)

In BT3 and BT4, subjects were put through the same perturbation regime as in the previous balance tests. In BT3, subjects received TSCS while in BT4 they did not. Perturbation force levels were the same, but the sequence of perturbation directions was randomized.

#### 2.1.7. Neurophysiological post-test (NT2)

Subjects were moved from RobUST onto a bed and made to lie supine for this part of the experiment. The same neurophysiological tests as in NT1 were carried out.

### 2.2. Measurements

#### 2.2.1. Variables calculated from balance test measurements

Motion capture data was collected during the balance tests with a nine-camera Vicon motion capture system (Vicon, Denver) and retro-reflective markers were placed bilaterally on the big toe, fifth metatarsal, lateral malleolus, heel, lateral femoral epicondyle, ASIS, PSIS, acromion, elbow and wrist. This marker set was used to generate the subject’s center of mass (COM) based on the method in Tisserand et al. (2016) and the anthropometric measurements in de Leva (1996). Ground reaction forces were collected with two 6-axis force plates (Bertec, Columbus, Ohio), one for each foot. The data from these two force plates were used to compute the foot center of pressure location. Both marker position and center of pressure data were smoothed with a 0.1 s wide moving average window.

##### 2.2.1.1. EMG data

Electromyography (EMG) data were collected bilaterally from 10 muscles, the soleus (SL), lateral gastrocnemius (LG), tibialis anterior (TA), bicep femoris (BF), and rectus femoris (RF). Muscle acronyms are prefixed with L or R in this paper for the left or right side of the body, respectively.

EMG signals were detrended, band-pass filtered between 20 and 300 Hz, full-wave rectified, and low-pass filtered at 20 Hz. The processed signal was used to calculate integrated EMG (iEMG) for each perturbation trial and the raw signal was used to calculate EMG activity duration (EMG_DUR).

iEMG was calculated as the summation of muscle activity over the perturbation trial and it was calculated and normalized using the formula:(1)

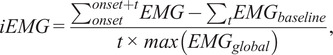

where 
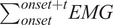
 is the summation of the EMG signal from perturbation onset over time *t* = 3 s window; and 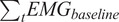 is the summation of a baseline EMG signal while the subject is in quiet standing over 3 s. 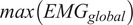 is the maximum observed EMG value per subject.

EMG duration was calculated from detrended EMG data passed through the Teager-Kaiser energy operator (TKEO) (Solnik et al., 2010), rectified and low-pass filtered at 50 Hz. First, an activation threshold was set using muscle activity data from a quiet standing recording taken at the beginning of the experiment. The threshold was set as the baseline’s TKEO output mean plus three standard deviations. A muscle was considered to be active when its TKEO signal rose above this activation threshold for more than 50 ms and inactive when the muscle signal fell below the threshold for more than 50 ms after being previously active. Activity duration was the sum of all intervals between muscle activations and deactivations.

Muscle coactivation was measured between the TA/SOL, TA/LG, and RF/BFagonist–antagonist pairs. Coactivation index was defined using as(2)

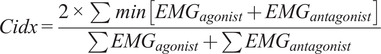



##### 2.2.1.2. Kinematic variables

Subjects’ performances were assessed using variables calculated from these measurements over a three-second duration after the initiation of a perturbation. Extrapolated center of mass (COM) and extrapolated margin of stability (MOS) were calculated as in Hof et al. (2005). The base of support (BOS) was bounded in the anterior–posterior direction by markers on the big toe and heel and in the lateral direction by markers on the right and left fifth metatarsals. The length of the vector from the COM to the COP (COPCOM) was assessed. The length of the path traced out by the COM, COP, and COPCOM during the three second period of the perturbation was recorded as that variable’s excursion. The maximum (Max), root mean square (RMS), and the timing relative to perturbation onset of the maximum (MaxIdx) of each of these variables and their first derivatives (VelMax, VelRMS, VelMaxIdx) were calculated.

##### 2.2.1.3. Balance strategies

Balance strategy refers to the use of either the ankle or hip strategy. We divided the body into an upper body trunk segment and a lower body segment (thighs, shank, and feet) and examined correlations between the upper body orientation in the sagittal plane and the lower body orientation. The ankle strategy was identified by positive correlations between the upper body and lower body while the hip strategy was identified by negative correlations between these segments (Blenkinsop et al., 2017). This gives us the classification for upper body and lower body rotations in Table 1.

**Table 1.** Results of correlation for different combinations of upper body and lower body rotations

	Anterior perturbation	Posterior perturbation
Ankle strategy	Trunk forward rotation – lower body forward rotation	Trunk backward rotation – lower body backward rotation
Hip strategy	Trunk forward rotation – lower body backward rotation	Trunk backward rotation – lower body forward rotation

Correlations were performed between the upper and lower body using a 250 ms centered moving window at each time point over the 3 s perturbation trial. The critical value/threshold for significance of *R* values for 25 data points (position data was recorded at 100 Hz) at 0.05 significance level is 0.3807. Subjects were said to be using the ankle strategy for *R* values above 0.3807, and the hip strategy for *R* values below −0.3807. *R* values between −0.3807 and 0.3807 were left undefined. Once strategies were identified, the percentage of the 3 s perturbation trial spent using the ankle strategy and the percentage time in hip strategy were calculated and recorded as Ankstrat and Hipstrat, respectively. These were used as the assessment variables.

##### 2.2.1.4. Ground reaction force

Ground reaction forces in the forward and backward directions were also recorded as GRF-APmax and GRF-APmin, respectively.

#### 2.2.2. Variables calculated from neurophysiological tests

Parameters generated from the H-reflex tests were derived from a sigmoid fit of the recruitment curve (Klimstra and Zehr, 2007). They include the maximum H-reflex response, Hmax, the maximum value of the slope of the sigmoid fit to the H-reflex response, HSlope, and the current that generates half of HMax, HCurrrent50.

Recovery cycle measurements produced the ratio of the conditioned H-reflex response to the non-conditioned response at 80, 150, 250, 500, and 1,000 ms ISIs. Finally, PRM reflex measurements produce the current threshold needed to excite PRMs in either the LSL or RSL muscles, PRM_Threshold, and the PRM reflex response of each muscle to 120% of the PRM_Threshold in NT1.

### 2.3. Statistical analysis

Prior to statistical analysis, failed perturbation trials were discarded, and the remaining trials were averaged separately for each perturbation direction. Paired *t*-tests were used to compare the performance variables measured in BT1, BT2, BT3, and BT4 to assess the effect of TSCS on balance. Paired *t*-tests were also used to compare the neurophysiological assessment variables in NT1 and NT2. No correction for multiple comparisons was made because of the small sample size. The significance level for all statistical tests was set to 0.05.

To assess the relationship between changes in the neurophysiological variables and the kinematic and EMG variables collected, Pearson correlation coefficients were computed between the percentage change in pairs of variables, and a Bonferroni correction was applied to adjust for multiple comparisons.

## 3. Results

The balance performance of subjects with and without continuous TSCS was compared, and the effect of a training intervention on these two conditions was also assessed. The results point to an increase in muscle activity accompanied by a decrease in balance proficiency with TSCS. All subjects completed the protocol, but some muscle activity data was lost from one subject and motion capture data from two subjects due to failure of the recording equipment.

A large number of comparisons were run in this experiment to assess subjects’ performance in the different conditions. This section will state the results, starting with the kinematic and EMG results from balance tests.

### 3.1. Immediate effects of stimulation on balance (BT1 vs. BT2)

**Figure 3.**
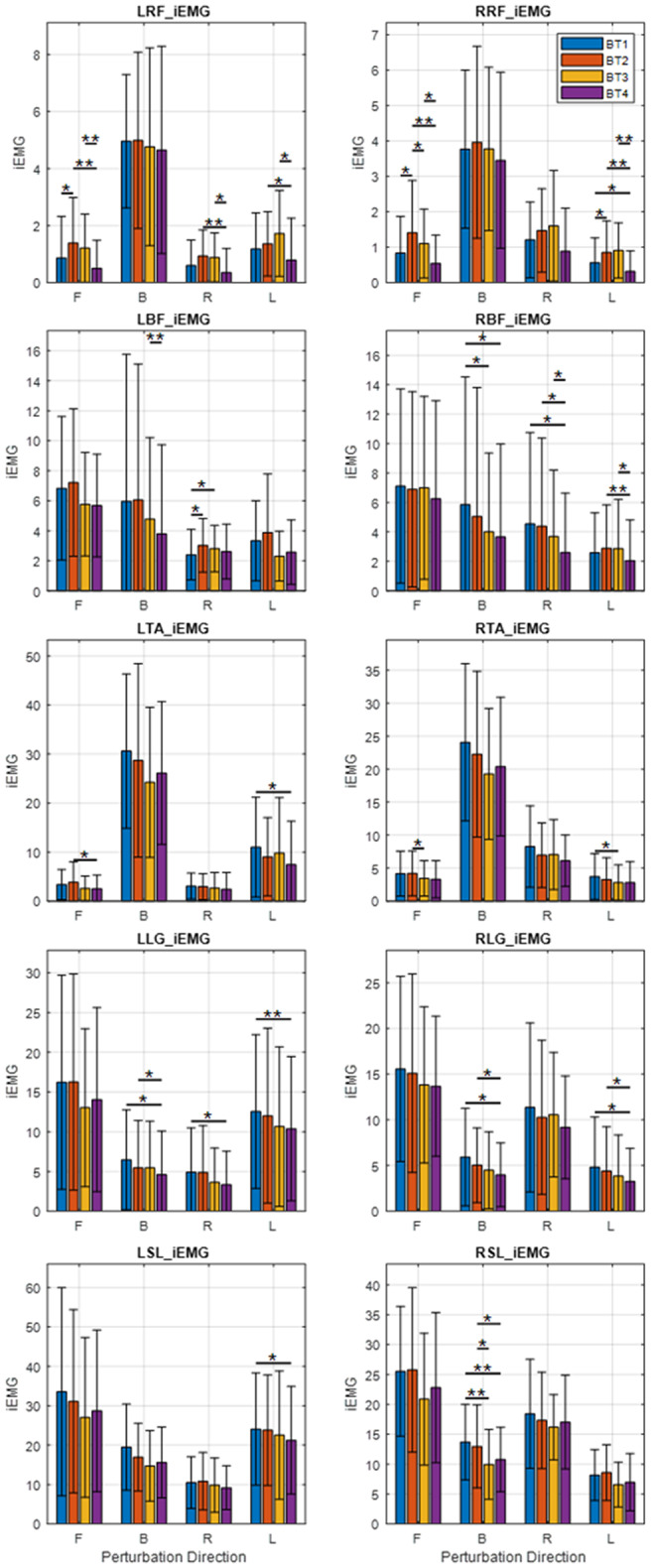
Average integrated EMG (iEMG) output of subjects in each direction (B, backward; F, forward; L, left; R, right). Significance bars represent the result of pairwise *t*-test comparisons between any two of BT1, BT2, BT3, or BT4. *p < .05; **p < .01.

A number of statistically significant changes were observed in the transfemoral muscles in multiple directions, but very few were observed in the transtibial muscles (Figure 3). All changes observed pointed to increased muscle activity. There was increased muscle activity in the L-RF for perturbations in the forward direction as evidenced by an increase in LRF_iEMG (*p* = .030) (Figure 3) while no statistical changes were observed in the other directions. There was a similar increase in muscle activity for forward perturbations observed in both RRF_iEMG (forward: *p* = .017) and RRF_Dur (forward: *p* = .045). An increase was also observed in RRF_iEMG for left perturbations (left: *p* = .033).

In the L-BF muscle, LBF_Dur showed increased muscle activity for forward and right perturbations (forward: *p* = .042, right: *p* = .032). There was also an increase in LBF_iEMG for right perturbations (right: *p* = .048). There was no significant change in RBF_Dur.

**Figure 4.**
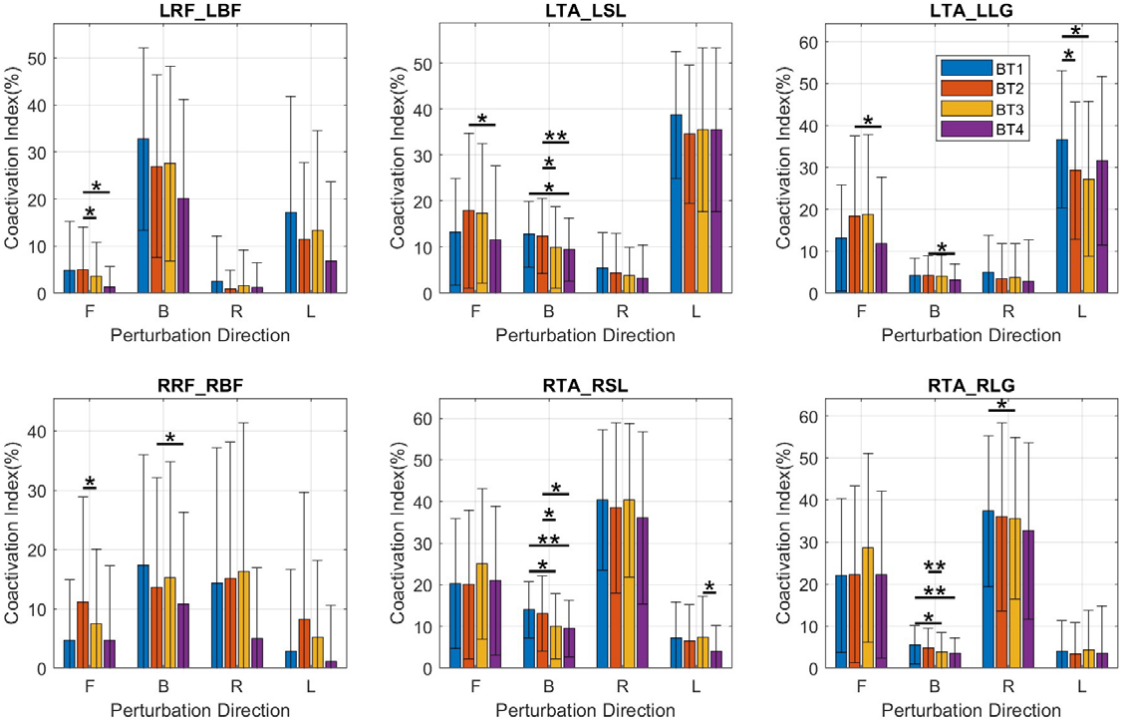
Bar plots of coactivation indices for muscle pairs. Mean and SD values are shown at BT1, BT2, BT3, and BT4 for perturbations in the forward (F), backward (B), right (R), and left (L) directions. BF, bicep femoris; L, left side; LG, lateral gastrocnemius; R, right side; RF, rectus femoris; SL, soleus; TA, tibialis anterior. 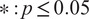 ; 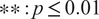 .

LTA/LLG CIdx decreased significantly only for left perturbations (left: *p* = .016) (Figure 4, top right). There were no significant changes observed in the L-TA/SOL, L-RF/BF, R-TA/LG, R-TA/SOL, and R-RF/BF CIdx’s.

**Figure 5.**
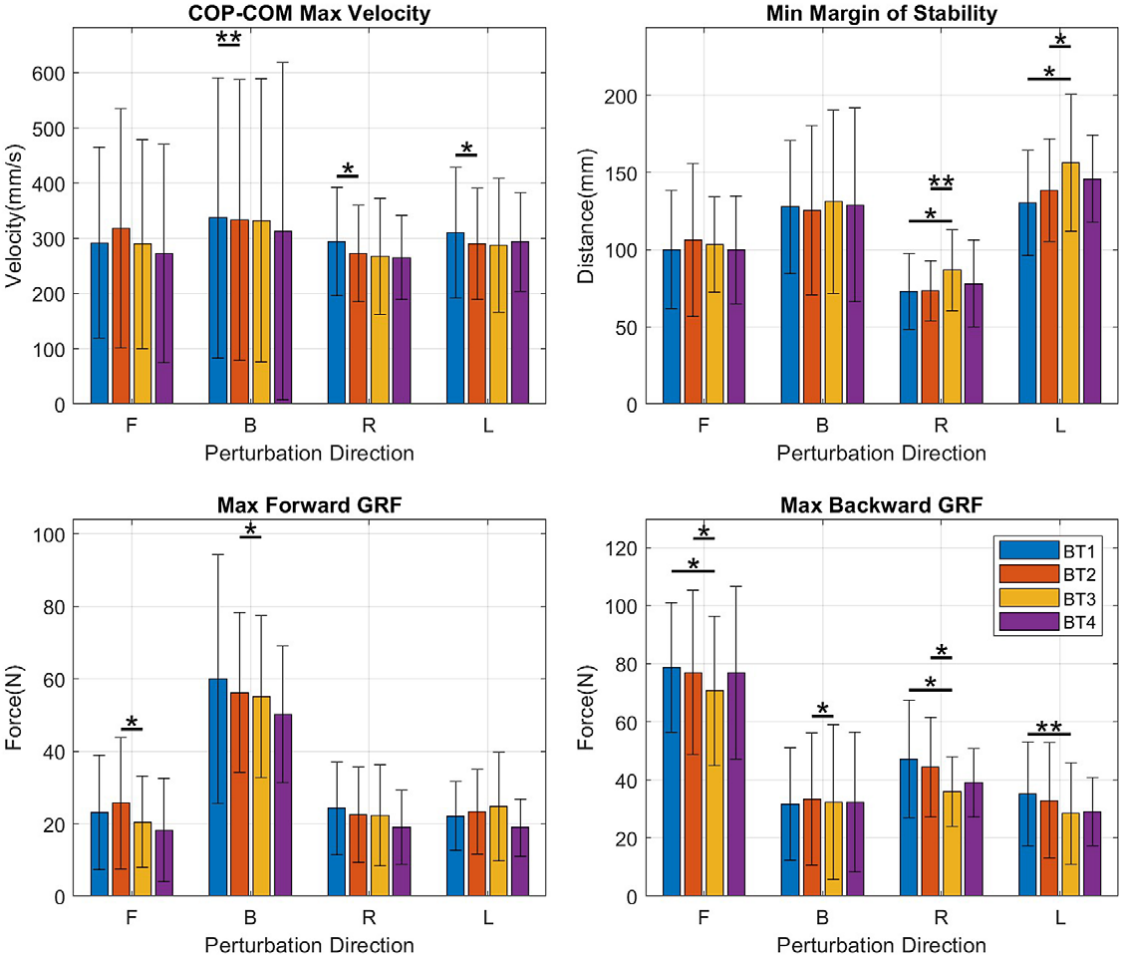
Bar graphs of kinematic measures – COP-COM max velocity, margin of stability, max forward ground reaction force (GRF), and max backward GRF – showing mean and SD values at BT1, BT2, BT3, and BT4 for perturbations in the forward (F), backward (B), right (R), and left (L) directions. 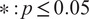 ; 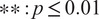 .

Among the kinematic measures (Figure 5), a small significant decrease in the MOS was observed only for forward perturbations (forward: *p* = .045) and was accompanied by a decrease in perturbation success rate (forward: *p* = .039), also only in the forward direction.

COPCOM VelMax, which can be seen as a measure of how quickly the subject reacts to a perturbation, decreased with significance in the backward, right, and left directions (backward: *p* = .006, right: *p* = .021 left: *p* = .024) but not in the forward direction in which no significant change was observed (Figure 5, top left).

### 3.2. Effect of virtual reality training (BT2 vs. BT3)

In the comparison of BT1 and BT2, muscle activity increases were observed in the transfemoral muscles but between BT2 and BT3 no such increases were observed. There were significant decreases in muscle activity in the LRF and RRF manifested by decreases in LRF_iEMG (forward: *p* = .028), RRF_iEMG (forward: *p* = .017) (Figure 3, row 1), and RRF_Dur (forward: *p* = .012). Decreases were observed in LTA_Dur (forward: *p* = .019), and RTA_iEMG (forward: *p* = .026) (Figure 3, row 3) for forward perturbations. In the backward direction, decreases were observed in RLG_Dur (backward: p = .002), and RSL_iEMG (backward: *p* = .031) (Figure 3, row 5). No significant changes were observed in the left and right directions.

Coactivation indices (CIdx) had decreases (Figure 4) in the LRF/LBF (forward: *p* = .041) and RRF/RBF (forward: p = .028) pairs and in the LTA/LSL (backward: *p* = .015), RTA/RSL (backward: *p* = .016) and RTA/RLG (backward: *p* = .007) pairs.

A decrease was observed in the maximum forward ground reaction force, GRF-APMax for perturbations in both forward (*p* = .043) and backward (*p* = .020) directions. This was accompanied by decreases in the maximum backward ground reaction force, GRF-APMin for perturbations in the forward (*p* = .028), backward (*p* = .039), and right directions (*p* = .035), suggesting a decrease in magnitude of the anteroposterior forces used in balancing in both directions (Figure 5, bottom-right). For both right and left perturbations, there was an increase in COM_Excursion (right: *p* = .020; left: *p* = .014) and in COP_RMS (right: *p* = .010; left: *p* = .019) which resulted in decreases in the MOS for both right and left perturbations (right: *p* = .008; left: *p* = .035).

### 3.3. Effect of training and TSCS after TSCS is terminated (BT1 vs. BT4)

Between BT1 and BT4, the balance tests at the beginning and end, there were significant decreases in activity observed in multiple muscles. Significant decreases in LLG_iEMG were observed in the backward, right, and left directions (backward: *p* = .013; right: *p* = .028; left: *p* = .010). There were decreases in RBF_iEMG, in the backward and right directions (backward: *p* = .012; right: *p* = .038); in RLG_iEMG in the backward and left directions (backward: *p* = .034; left: *p* = .029); in RLG_Dur in the backward and left directions (backward: *p* = .002; left: *p* = .024); in the RRF_iEMG, in the left (*p* = .026) direction; in RSL_iEMG (backward: *p* = .004) and in RSL_Dur (backward: *p* = .002) (Figure 3).

Decreases were observed in the LTA/LSL CIdx (backward: *p* = .046) (Figure 4, top-middle), RTA/RSL CIdx (backward: *p* = .008) (Figure 4, bottom-middle), and RTA/RLG CIdx (backward: *p* = .004) (Figure 4, bottom-right).

Notably, no significant changes were observed in the kinematic and EMG measures for forward perturbations and no notable changes were observed in the kinematic variables in all directions.

### 3.4. Effect of training and TSCS (BT1 vs BT3)

Increases in muscle activity were observed in some muscles, evidenced by an increase in LBF_iEMG (right: *p* = .013) and RRF_Dur (backward: *p* = .042). Other muscle activity changes were decreases in LSL_Dur (backward: *p* = .008), RLG_Dur (backward: *p* = .006), RSL_Dur (backward: p < .001), RSL_iEMG (backward: *p* = .003) and RTA_iEMG (left: *p* = .037).

There were decreases in LTA/LLG Cidx (left: *p* = .045), RTA/RLG (right: *p* = .028) and RTA/RSL (backward: *p* = .020) and RTA/RLG (backward: *p* = .026) (Figure 4).

A decrease was observed in MOS (backward: *p* = .020) for backward perturbations but was not observed for perturbations in other directions.

### 3.5. BT2 vs. BT4

There were no significant muscle activity increases between BT2 and BT4, all significant changes being decreases. Decreases were observed in LBF_Dur for forward and backward perturbations (forward: *p* = .041; backward: *p* = .014); in LRF_Dur for backward (*p* = .018) perturbations; in LLG_iEMG for backward (*p* = .012) perturbations, LRF_iEMG for backward, forward, right and left perturbations (forward: *p* = *p* = .014; backward: *p* = .002; right: *p* = .005; left: *p* = .028); LTA_Dur for forward and right perturbations (forward: *p* = .005; right: *p* = .050); and in LTA_iEMG for forward (*p* = .016) perturbations. On the right side, decreases were observed in RBF_Dur for right and left perturbations (right: *p* = .018; left: *p* = .045); RBF_iEMG for right and left perturbations (right: *p* = .022; left: *p* = .006); RLG_Dur for backward and left perturbations (backward: *p* = .002; left: *p* = .008), for RLG_iEMG for backward and left perturbations (backward: *p* = .013; left: *p* = .028; for RRF_iEMG for forward and left perturbations (right: *p* = .004; left: *p* = .005); and RSL_iEMG for backward (*p* = .013) perturbations (Figure 3).

LTA/LLG CIdx decreased in the forward (*p* = .014) and backward (*p* = .035) directions, LTA/LSL CIdx in the forward (*p* = .012) and backward (*p* = .002) directions, RRF/RBF in the backward (*p* = .042) direction and RTA/RSL in the backward (*p* = .021) direction (Figure 4).

### 3.6. H-reflex recruitment curve analysis

The H-reflex recruitment curve was fit to 10 of the 11 subjects at *R*^2^ error above 90%. The number of data points used to fit the recruitment curve ranged between 6 and 12. The *R*^2^ error measuring goodness of fit had a mean = 0.971, SD = 0.029. With the curves fit and the curve characteristics extracted, paired t-tests were carried out between results in NT1 and NT2. No significant changes between NT1 and NT2 were observed in HMax (*p* = .874), HSlope (*p* = .198), and HCurrent50 (*p* = .542).

### 3.7. PRM results

A *t*-test comparison of the PRM reflex threshold at NT1 and NT2 yielded no significant change (*p* = .864). There was no uniform effect of the protocol on the subjects as PRM_Threshold increased by 3 mA or more in two subjects, decreased by 3 mA or more in four others, and changed by less than 3 mA in five subjects.

Subjects’ responses to 120% of the spinal stimulation threshold established in NT1 did not show any significant change between NT1 and NT2.

### 3.8. H-reflex recovery cycle

*t*-test comparisons between the magnitude of the pretest and posttest conditioned H-reflex at 80, 150, 250, 500, and 1,000 ms ISIs yielded a significant increase only at the 500 ms ISI (*p* = .04).

### 3.9. Correlation between changes in variables

When we observed the strategies used by subjects, we noticed that they reacted in two distinct ways. As shown in Figure 7, all subjects started out applying an ankle strategy but about 500 ms after the start of the perturbation, subjects 3, 6, 10, and 11 turned to using the hip strategy while the other subjects did not. Based on this observation, the subjects were divided into two groups. An ANOVA was carried out to examine the effect of two factors: (i) the training intervention (i.e., the effect of the protocol) and (ii) the group (i.e., the balance strategy used). The ANOVA confirmed that the balance strategies employed by the two groups were significantly different *F*(17.758, *df* = 1) = *p* < .001), but there was no effect of training nor an interaction effect between training and group. It was also found that the strategy had a strong correlation with the change in HCurrent50 (*R*(7) = .7798, *p* = .013) between NT1 and NT2 One group had a positive percentage change and the other group a negative. They shall be called 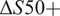 and 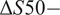 , respectively. Along these lines, group differences were observed in other variables as well. COP_Excursion (*F*(1,7) = 5.928, *p* = .022) COP_Vel (*F*(1,7) = 5.703, *p* = .24), COPCOM (*F*(1,7) = 9.366, *p* = .005), COPCOM_Vel (*F*(1,7) = 6.886, *p* = .014).

Examining the difference in performance between 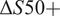 and 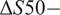 groups during each balance test, it was observed that there were significant differences in forward perturbation in the following: Hipstrat at BT1 ( 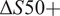 = 53.36 ± 4.61% vs. 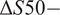 =44.83 ± 4.20% *t*(6.247) = −2.868, *p* = .027), and BT3 ( 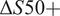 = 54.64 ± 4.33 vs. 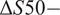 = 39.01 ± 4.52%, *t*(6.699) = −5.28, *p* = .001) with the 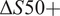 group favoring the hip strategy. With respect to muscle activity, there were no significant differences at BT1 and BT2 but significant differences were present in BT3 for RRF_Dur (1.96 ± 1.31 s vs. 0.25 ± 0.22 s *p* = .042), LRF_Dur (1.55 ± 0.20 s vs. 0.18 ± 0.20 s, *p* = .012) and LRF_iEMG (*p* = .005) and in BT4 for RRF_Dur (1.39 ± 0.69 s vs. 0.20 ± 0.23 s, *p* = .020), LRF_Dur (0.88 ± 0.46 s vs. 0.11 ± 0.00 s, *p* = .020), LRF_iEMG (*p* = .027) with the 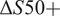 group having higher muscle activity.

## 4. Discussion

This experiment aimed to explore the effect of TSCS applied during the performance of a functional task. The experiment tested the impact of TSCS on performance immediately after it was activated and its effects when TSCS was combined with functional training. It was hypothesized that exposure to TSCS would increase spinal excitability, which could have an incremental effect on reactive balance control. It was also hypothesized that TSCS would increase background muscle activation levels, facilitating higher muscle exertion during a task. Muscle activation increases were observed in some conditions (particularly between BT1 and BT2), but a decrease in perturbation success rate accompanied these increases.

It is a general rehabilitation principle that postural strategies improve with repeated practice of a movement (Horak et al., 1997; Kleim and Jones, 2008). As subjects become more accustomed to a disturbance, the reactions involve less exaggerated muscle activation and an improvement in performance (Horak et al., 1997). Welch and Ting (2014) and Babič et al. (2016) explain that these changes result from the motor system optimizing for safety, energy expenditure, and task requirement. Thus, as subjects become more comfortable and familiar with a task, they make a tradeoff between energy expenditure and safety while still fulfilling the task requirement. This effect was observed as muscle activity reduced in some conditions of the experiment, and it will be referred to here as the practice adaptation effect.

### 4.1. Spinal excitability

**Figure 6.**
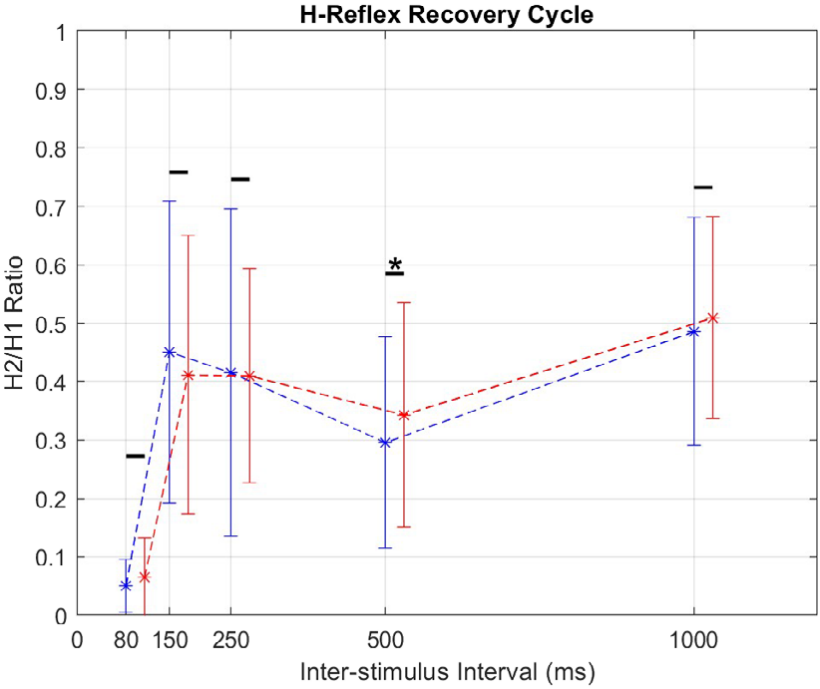
Graph of H-reflex ratio against inter-stimulus interval (ISI). The ratio, H2:H1, is the ratio of the second to the first H-reflex response to a paired-pulse averaged for all trials of a subject and then over all subjects. Blue points are from the pretest, NT1, and red points are from NT2. 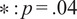 .

Assessments of the H-reflex recruitment curve, its recovery cycle, PRM threshold, and PRM amplitude responses did not yield significant results except for an increase in the conditioned amplitude at 500 ms ISI. The dominant process that causes attenuation of the H-reflex at this stage of the recovery cycle is homosynaptic depression (Clair et al., 2011; Hofstoetter et al., 2019) and it is said to be caused by a reduced probability of neurotransmitter release after the initial activation at the synapse. The small recovery increase in H-reflex recovery observed at 500 ms (Figure 6) could be explained as the priming of the spinal circuits after being stimulated for an amount of time and can be seen as the system being primed for activity.

**Figure 7.**
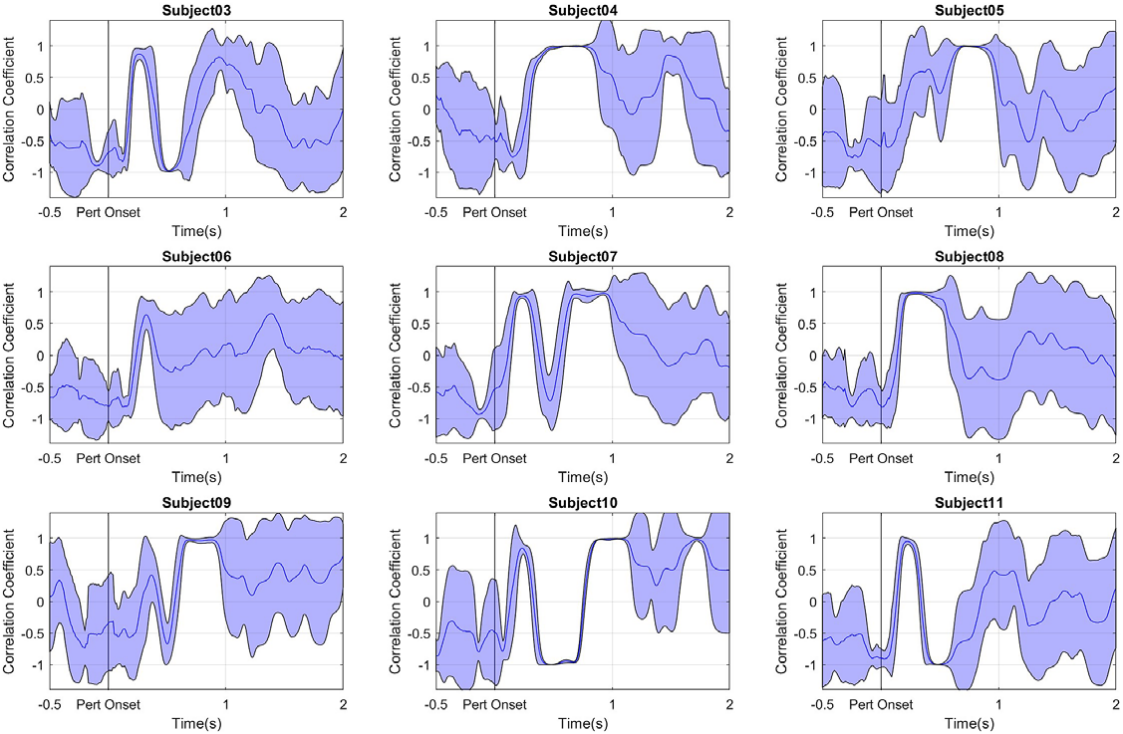
Correlation coefficient of trunk rotation and shank rotation. Positive values above 0.3807 indicate Ankle strategy and negative values below −0.3807 indicate Hip strategy. Subjects 4, 5, 6, 8, and 9 have average correlation coefficient significantly lower than subjects 3, 7, 9, 10, and 11 indicating that they use the hip strategy more often.

The lack of significant changes in the other variables may be due to the timing of the assessments. Between the end of BT3, when TSCS was deactivated, and the start of NT2, the neurophysiological post-test, BT4 was carried out. This took 30 min on average. It is possible that the effects of TSCS had worn off before the start of NT2, and spinal excitability changes were not captured with our measurement protocol.

In Lamy et al. (2012) and Carrera et al. (2022), TSCS was shown to increase H-reflex recovery and also increase the synaptic efficacy of the afferent—motoneuron connection. Therefore, we would expect a similar increase in recovery in this experiment, but this was not the case. Hence, alternatively, the lack of neurophysiological changes may be due to an interaction between two effects: the excitatory effect of TSCS and the practice adaptation effect of training.

### 4.2. Immediate effects of TSCS (BT1 vs. BT2)

The comparison between BT1 and BT2 best demonstrates the effect of TSCS on muscle activity in this experiment. TSCS led to an increase in muscle activity in select muscles in the forward, left, and right directions. However, the increase in the forward direction was accompanied by a decrease in balance performance. These results were presented previously in Carrera et al. (2022). It is important to note that these increases occurred after BT1. We have seen in previous experiments and in the literature that muscle activity reduces with repeated activity (Horak et al., 1997) and so, despite BT2 occurring after BT1 this muscle activity increase was still observed. Our observation of increased muscle activity is thus unusual and suggests an effect of TSCS.

Also, noteworthy is the observation that where increases in muscle activity were observed for perturbations in a certain direction, the increases occurred in muscles that would ordinarily not be used to react to these perturbations. This suggests that the central nervous system (CNS) prioritizes the control of goal-critical muscles by making them conform to the activations required for the task at hand and suppressing the effect of TSCS apparent in the non-critical muscles.

The observed muscle activity increases were accompanied by reduced balance performance. In fact, the trial success rate and the average MOS for perturbations in the forward direction decreased with the use of TSCS. The increase in muscle excitation engendered by TSCS and the accompanied deterioration of balance performance suggests TSCS causes a disruption in movement coordination. This disruption could be a result of TSCS interfering in inhibitory processes in the CNS as it promotes excitation of sensory neurons. Inhibitory processes serve to prevent excessive activation of motoneurons by inhibiting the signal from sensory neurons (Wilson, 1966; Thayer, 2006). Previous studies by Lamy et al. (2012) and Carrera et al. (2022) showed increased efficacy of the sensory neuron–motoneuron connection after TSCS. If we assume that this same effect occurred in this experiment, it would mean that subjects experienced increased triggering of the motoneurons. We can conclude then that this increased motoneuron triggering was disruptive which for healthy subjects is understandable as their balance system already works optimally.

### 4.3. Effect of training with TSCS (BT2 vs. BT3)

The overarching change observed between BT2 and BT3 was a reduction in muscle activity and an adaptation towards the use of less force in reactive balance control. Subjects had TSCS in both tests, but BT3 occurred after the training intervention. The subjects, thus adapted to the task as a result of training leading to decreased muscle activity. In the forward direction, decreases were observed in the L-RF and R-RF muscles, the same muscles in which increased activity was observed between BT1 and BT2. These reductions, accompanied by a non-significant increase in perturbation success rate (93% in BT2 to 97.5% in BT3), suggest that the neural system of the subjects adjusted to TSCS input and reduced muscle activity to levels that allowed for the restoration of the initial level of balance performance. The increased success rate, decreases in ground reaction force and MOS, and increase in COM_Excursion suggest that after the training intervention, the subjects arrived at a more successful strategy for balance control that required less vigorous reactions. Therefore, the adaptive processes of the motor system were able to overcome the disruptive effect of TSCS, suppress the heightened muscle activity, and achieve a higher level of performance.

### 4.4. Effect of deactivating TSCS (BT2 vs. BT4)

The forward direction was where the LRF and RRF experienced increases between BT1 and BT2 (TSCS effect) and decreases between BT2 and BT3 (practice adaptation effect). Along with other muscles, muscle activity reductions were observed in the LRF between BT3 and BT4 in all directions. This muscle, which most exhibited activity increasing effects of TSCS displayed a large fall in muscle activity once TSCS was deactivated, suggesting that TSCS increased muscle activity in previous balance tests. The activity reduction effect also contributed to the reduced activity observed in the LRF and RRF muscles.

Between BT2 and BT4, there is the double pressure of the activity reduction effect and the removal of TSCS. These results show that large muscle activity decreases strongly point towards the counterbalancing of these effects.

### 4.5. Effect of training and TSCS (BT1 vs. BT4)

In comparing BT1 and BT4, we tested whether there were any residual effects of TSCS and the training intervention after TSCS was deactivated. At this point, the subject had been subjected to what we think are the opposing effects of practice adaptation and the activity augmenting effect of TSCS. TSCS was not used in both tests. In the right, left, and backward directions muscle activity decreases were observed in a large number of muscles which may have been caused by the doubly depressing effect of the removal of TSCS and the practice adaptation effect.

In the forward direction, there were no significant increases or decreases. In an experiment without TSCS it would be expected that the practice adaptation effect would have caused muscle activity reductions but its absence points towards a residual effect of TSCS. The presence of large muscle activity reductions in the other directions would suggest that TSCS did not have an effect on muscle activity and only the practice adaptation effect was at play. An alternative explanation that builds on our expectations of what the effect of TSCS should be can be proposed. Instead of the increase in excitatory stimuli introduced by TSCS, as observed between BT1 and BT2, we hypothesize that the motor system adapts by suppressing this new excitation experienced in the body. The body moves towards suppression because it would seem that increased excitation in healthy subjects is disadvantageous as we observed a decrease in perturbation success rate for perturbations in the forward direction. So, in order for the motor system to continue to perform optimally this excitation is suppressed. Both the inhibitory processes in the spine and the descending supra-spinal commands are capable of causing this suppression. Hence, it would seem that the adaptation induced by TSCS in the subjects is not the muscle activity increase hypothesized but a stronger suppression of muscle activity.

### 4.6. Other results

One peculiar result worth investigating in future studies is the presence of a relationship between the percentage change in HCurrent50 of the H-reflex and the balance strategy utilized by the subjects. When subjects were grouped into 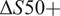 and 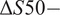 , we found that 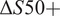 used the hip strategy significantly more in BT1, BT3, and BT4, while 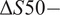 used the ankle strategy more. As this bifurcation was not the result of TSCS, it is suggested here that there is a relationship between the subjects’ tendency to select the ankle or hip strategy and their neurophysiological reaction to stimulation.

With respect to muscle activity, there were no significant differences at BT1 and BT2 but significant differences were present in the RRF and LRF between BT3 and BT4 with the 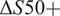 group having higher muscle activity.

By definition, the 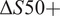 group experienced a right shift of the H-reflex recruitment curve implying that more current was needed to generate the same H-reflex response, further implying that the reflex became less excitable. The reverse could also be inferred, i.e., that the 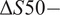 group’s H-reflex became more excitable. Therefore, subjects who favored the hip strategy, which involves greater coordination (Harsha and Sureban, 2012) and who displayed greater muscle activity, at the end of the training, adapted towards suppression of the H-reflex, a right shift of the recruitment curve. Vice versa, subjects who favored the ankle strategy adapted towards greater excitability of the H-reflex. We can further infer from this that more muscle coordination leads to more suppression. At this point of our research, we can only speculate about the cause of this relationship but future experiments can be set up to determine the veracity and significance of this finding.

### 4.7. Conclusion

In this study, we have shown the effect of applying TSCS during a training exercise in healthy subjects. Most significantly, we observed the interaction of the muscle activity reduction effect caused by continuous repetition of an activity and the muscle activity augmentation effect of TSCS. Once TSCS is turned on there is an initial increase in muscle activity in select muscles which leads to worse perturbation performance in the forward direction. Subsequently, with more exposure to the perturbations and after training, the motor system adjusts and returns muscle activation levels lower and coordination improves. With the removal of stimulation, muscle activity gets even lower, suggesting that the body has adapted toward reducing muscle activity. The mechanism of this reduction was not investigated but was hypothesized to be greater presynaptic inhibition, hyperpolarization of the sensory afferents, or the blocking of natural sensory inputs.

These results provide insight into the complex interactions of different elements in the CNS and what triggers could be used to get a desired behavior. In future studies, the activity combined with TSCS should be one in which greater muscle exertion leads to better performance in order to take advantage of the effect of TSCS. Alternatively changing the parameters for TSCS could generate different adaptations that could lead to improvements in task performance. For example, 60 Hz stimulation has been used to reduce spasticity. This could generate increased inhibition in healthy subjects as well which could possibly lead to greater control and better performance. Nevertheless, even though the 30 Hz monophasic pulsed current TSCS used in this experiment did not improve task performance, it did result in increased spinal excitability and increases in muscle activity. These effects could be beneficial for SCI as the challenge with SCI patients is to increase motor neuron activity below the lesion. Cortico-spinal inhibitory processes which limit and control the firing of motor neurons in healthy subjects are impaired in SCI (Benedetti et al., 2022) and will be one less source of interference with the effects of TSCS. TSCS, thus, may have more of an effect on SCI subjects. The use of TSCS with SCI will be the subject of future investigations.

## Data Availability

Derived data supporting the findings of this study are available from the corresponding author (I.O.) on request.
